# Seismic source identification of the 9 November 2022 M_w_ 5.5 offshore Adriatic sea (Italy) earthquake from GNSS data and aftershock relocation

**DOI:** 10.1038/s41598-023-38150-5

**Published:** 2023-07-16

**Authors:** G. Pezzo, A. Billi, E. Carminati, A. Conti, P. De Gori, R. Devoti, F. P. Lucente, M. Palano, L. Petracchini, E. Serpelloni, S. Tavani, C. Chiarabba

**Affiliations:** 1 Istituto Nazionale di Geofisica e Vulcanologia, Osservatorio Nazionale Terremoti, Rome, Italy; 2 Consiglio Nazionale Delle Ricerche, IGAG, Rome, Italy; 3 Dip. Scienze Della Terra, Sapienza Università di Roma, Rome, Italy; 4 Istituto Nazionale di Geofisica e Vulcanologia, Osservatorio Etneo, Catania, Italy; 5 Istituto Nazionale di Geofisica e Vulcanologia, Sezione di Bologna, Bologna, Italy; 6 DISTAR, Università Degli Studi di Napoli “Federico II”, Naples, Italy

**Keywords:** Geophysics, Seismology, Tectonics

## Abstract

The fast individuation and modeling of faults responsible for large earthquakes are fundamental for understanding the evolution of potentially destructive seismic sequences. This is even more challenging in case of buried thrusts located in offshore areas, like those hosting the 9 November 2022 Ml 5.7 (M_w_ 5.5) and M_L_ 5.2 earthquakes that nucleated along the Apennines compressional front, offshore the northern Adriatic Sea. Available on- and offshore (from hydrocarbon platforms) geodetic observations and seismological data provide robust constraints on the rupture of a 15 km long, ca. 24° SSW-dipping fault patch, consistent with seismic reflection data. Stress increase along unruptured portion of the activated thrust front suggests the potential activation of longer portions of the thrust with higher magnitude earthquake and larger surface faulting. This unpleasant scenario needs to be further investigated, also considering their tsunamigenic potential and possible impact on onshore and offshore human communities and infrastructures.

## Introduction

**Figure 1:**
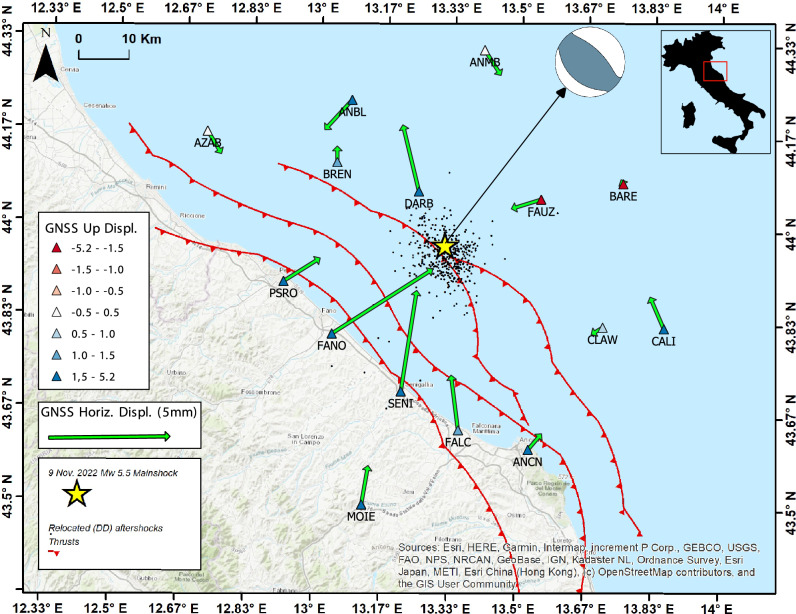
Epicentral area of the 9 November 2022 offshore earthquake (yellow star). Black dots are the aftershocks. Green arrows are the horizontal coseismic displacements recorded by the GNSS stations (triangles); tringle colors indicate the vertical coseismic displacement as indicated in legend. We also report the main thrusts^6^ close to the seismic sequence and the moment tensor calculated for the mainshock (http://terremoti.ingv.it/event/33301831).

On 9 November 2022, a M_L_ 5.7, (M_w_ 5.5) earthquake occurred about 30 km offshore the Marche region in the Adriatic Sea (Fig. 1). The hypocenter-coast distance preserved human lives and infrastructures, limiting the damage.

A minute after the mainshock, a large (M_L_ 5.2) shock occurred about 8 km south of the first event (http://terremoti.ingv.it/). Hypocentral determination of offshore events suffers from the poor azimuthal coverage of the seismic network, and the large distance of seismic stations installed along the coastline. Thus, the correct recognition of the seismic source requires a multidisciplinary approach and the use of different (geological and geophysical) datasets. The sequence is located in correspondence of the Northern Apennines frontal thrust systems, along which ongoing convergence is accommodated, with strain-rates of the order of 30–50 nanostrain/yr^1,2^, on a suite of different and sub-parallel buried faults still poorly addressed^3–6^.

The mainshock moment tensor solution, calculated using the Time Domain Moment Tensor technique^7^, indicates a reverse mechanism on a NW–SE trending fault plane (Fig. 1) (http://terremoti.ingv.it/event/33301831), as well as the moment tensor solutions of the M_L_ 3.5 + events of the sequence. No moment tensor solution has been computed for the M_L_ 5.2 event, due to the overlap and interference of phases from the two events. Time–space aftershocks distribution after the mainshock occurrence is crucial to understand the activated geological structures, the role of fluids as soon as the evolution of the sequence. Thus, we analyzed data from the INGV seismic control room to characterize seismicity in terms of magnitude distribution and decay with time. We also relocated the 9 November earthquake and aftershocks by using the hypoDD relative method^8^ lighting a well-defined fault plane. In seismic source identification, a primary role is fulfilled by the coseismic displacement field reconstruction. However, the detection of ground deformation signals represents a significant issue, because of the offshore location of the sequence. Available GNSS stations covering the coastal area (Fig. 1) show significant coseismic offsets in relative time-series, which sum the cumulative deformation of the two M > 5 seismic events (in Fig. S1 of Auxiliary materials we report the SENI and FANO time series example). Taking advantage of some onshore (storage centers) and offshore (seabed-anchored hydrocarbon platforms) industrial infrastructures hosting continuous GNSS stations, integrated with public and private GNSS networks onshore, we collected a precious dataset to infer additional constraints on the activated buried thrust. This is the first time that GNSS observations collected on offshore infrastructures are used to constrain the coseismic deformation pattern of an earthquake. Offshore platforms generally stand on legs of steel or concrete that are piled into the sea floor, and thanks also to their own weight remain fixed in place, securely anchored to the sea floor. Their stability has been also proven by the long-term GNSS time-series acquired by on-board continuous stations^9,10^ therefore they are suitable for active tectonic studies^1^. Here, by processing the extensive dataset collected at both onshore and offshore GNSS stations, we estimated the cumulative coseismic deformation pattern related to the 9 November 2022 seismic events. Despite its millimeter-scale, the coseismic displacement field clearly depicts a reverse faulting mechanism pattern, providing key constraints for the seismic source identification (Fig. 1) and subsequent stress transfer analysis.

In this study, we show a fast preliminary space–time characterization of the seismic sequence obtained by seismic data from the INGV monitoring system and the coseismic slip as constrained by GNSS data, coupled with available subsurface public seismic reflection data. Our main aim was to constrain the source of the 2022 Adriatic earthquake for its use in future studies of different kinds (e.g., hazard mitigation, earthquake and tsunami scenarios, hydrocarbon platform safety).

## Geological setting

In the northern Adriatic Sea, the external portion of the Apennine orogenic belt involves the sedimentary succession of the Adria passive margin and the overlying foredeep deposits associated with the Neogene to recent W‐directed Apennines subduction^11^. In this sector of the Apennines orogen, the thrust front is buried beneath Early Pliocene‐Quaternary synorogenic deposits, as indicated by seismic‐reflection data^6,12–15^. The thrust system is characterized by ramps climbing off the basal detachment, located within the Triassic evaporites, to join upward a shallower detachment located along Early Miocene marls^1,15,16^. Several seismic sources were associated with these structures, as also suggested by instrumental and historical seismicity^5,17^. Recently released GNSS data from offshore hydrocarbon seabed-anchored platforms, located few tens of kilometers from the epicentral area, permitted to define a shortening rate of about 1.5 mm/yr^2,9^ mostly accommodated along the Apennines frontal thrusts in a SW-NE direction^1^, significantly improving our knowledge of the shortening rate accommodated between the Northern Apennines and the Adria plate^18,19^.

**Figure 2:**
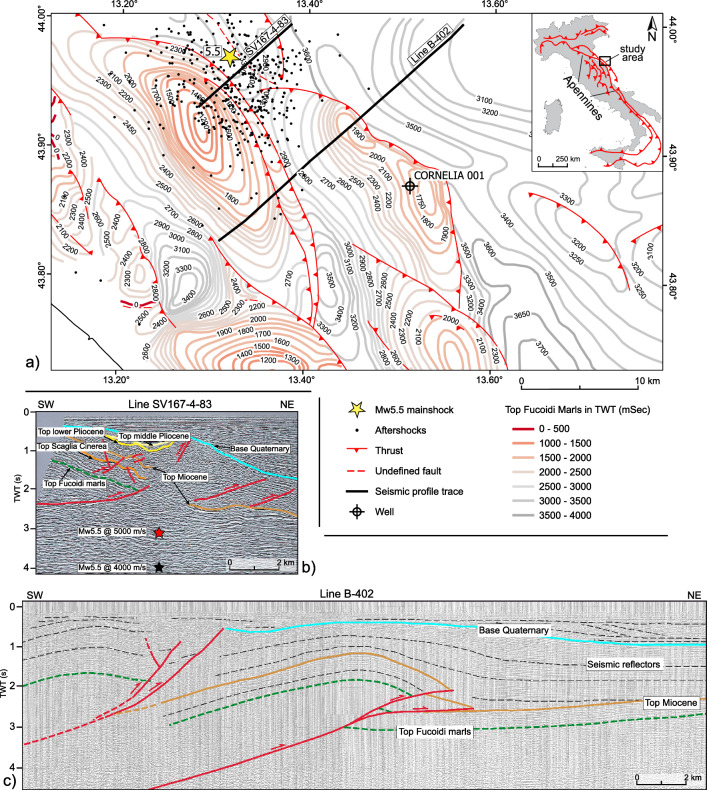
Thrust system of the area. (**a**) Subsurface structural framework highlighted with the isochrone map of the Fucoidi Marls Fm. (Aptian-Albian) providing a first order information of the geological context and a general overview of the main thrust fronts in the area. The map is in two-way times (ms) with the datum plane set at 0 m a.s.l. (mod. after^20^). The location of Cornelia 1 well available in the VIDEPI project (http://www.videpi.com) is reported. The thick black lines show the location of the B-402 and the SV-167-4-83 seismic profiles reported below (http://www.videpi.com). (**b**) Redrawing from public database (http://www.videpi.com) of the SV-167-4-83 seismic profile (location in **a**). A fault-related fold is clearly visible, but any interpretation of the fault geometry and the related depth conversion is affected by the high noise versus signal ratio of the seismic dataset (particularly below 2 Sec TWT). The hypocenter mainshock (M_w_ 5.5) as computed in this work (i.e., 8 km) has been converted in time using two end-member velocity values for the entire sedimentary package (i.e., 4000 m/s and 5000 m/s). The results indicate that the hypocenter of the mainshock is likely to be correlated to the activity of the more external Apennine thrust front. (**c**) Interpretation of the B-402 seismic profile (location in **a**) showing the upper portion of the most external thrust deforming the Meso-Cenozic carbonate succession. Line B-402 provides an example of fault geometries in the area. The Cornelia well and public isochrone maps (http://www.videpi.com) have been used to calibrate seismic horizons in B-402 profile. We report the thrust fronts in map view^20^.

The frontal sector of the Northern Apennine orogen is characterized by NW–SE striking and NE-verging double-plunging anticlines developing above SW-dipping ramps (Fig. 2). The November 2022 seismic sequence is located along the most external structure of the Apennine orogen^20–22^. In the epicentral area, only poor-quality vintage public seismic profiles are available, and it hazardous, to discriminate fault planes at depth (Fig. 2b). We tentatively plotted the time-converted hypocenter along the SV-167-4-83 seismic profile (http://www.videpi.com) using two end-member constant velocity scenarios (i.e., 4000 m/s and 5000 m/s) to highlight the possible depth range of the mainshock hypocenter (Fig. 2b). Seismic line B-402, located 10 km far from the mainshock and available from ViDEPI public database (http://www.videpi.com), provides a general characterization of the structural setting of this frontal sector of the Northern Apennine (Fig. 2c). Line B-402 allowed the recognition of a flat-ramp-flat geometry for the external thrust, with the lower flat not clearly detectable but interpreted to be deeper than 4 s TWT (Fig. 2c).Considering the hypocentral depth of the mainshock and aftershocks, we suggest that the activated portion of the thrust corresponds to the ramp, intermediate between the deeper and shallower detachments, and involves the Mesozoic carbonate sequence, likely down to the Triassic evaporites.

## Results

### Earthquake locations and seismological parameters

**Figure 3:**
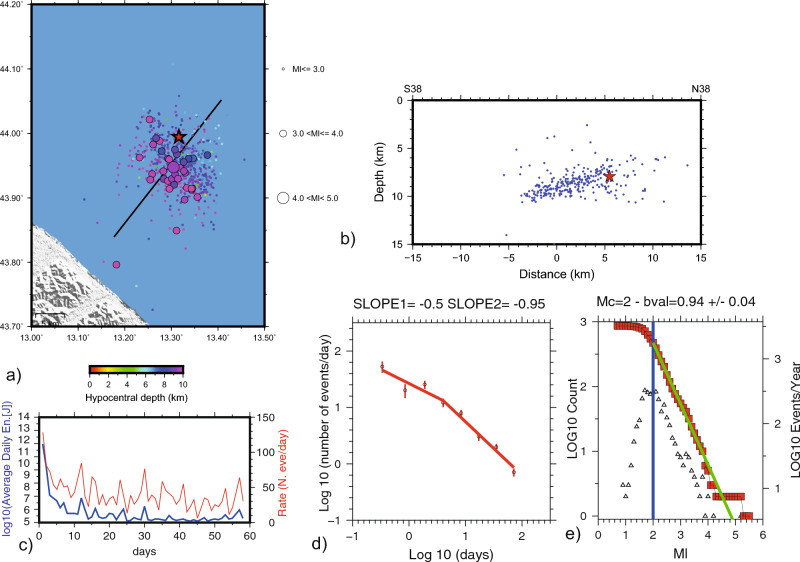
DD aftershock relocation. (**a**) Map view of the relocated aftershocks; we also show the profile traces of panel (**b**) where we report events in a 3 km wide buffer; in both panels, star represents the M_w_ 5.5 mainshock. (**c**) Daily earthquake rate (red line); in blue the daily average energy derived by Ml^44^. (**d**) The number of aftershocks/day as a function of the time elapsed from the mainshock: it is best fitted by a two-slope model (with a likelihood of 55%) than a single-slope Omori-like behavior (not shown). The slope of the first and second segments is indicated on top of the panel. (**e**) The Gutenberg and Richter aftershock distribution for Mc = 2 shows a b-value of 0.941 ± 0.044.

A total of 594 aftershocks in the period 22 November 2022–05 January 2023 and the main shock have been relocated by using the hypoDD relative method^8^ on *P-* and *S-*wave arrival times picked by the seismologists on duty in the INGV control room. Results differ from those of the INGV control room (available at http://terremoti.ingv.it/): in particular, the M_w_ 5.5 is located about 4.4 km south and 4.1 km deep with respect to the control room one. We estimate the uncertainties on the location parameters through a bootstrap approach (see the Methods section for the details on the location procedure and error estimation). Aftershock relocations (Fig. 3) show a NNW-trending, 25° SSW-dipping, 15 km long fault, seismically defined between 5 and 10 km depth. The original data from the INGV seismic control room have been analyzed to characterize the magnitude distribution and time decay of the aftershocks. We estimated magnitude distribution of the seismic sequence following the Gutenberg-Richter^23^ recurrence relation. The inferred magnitude of completeness (Mc) value is rather high (Mc = 2.0), as expected for the large distance to the coast (15–20 km) and the high noise of the closest stations installed on thick sediments of the Adriatic foreland, and the b-value is 0.94 (Fig. 3).

We plot the number of aftershocks/day and the daily average energy release as a function of time, above the completeness magnitude threshold of 2.0, which is reached 4 h after the mainshock (Fig. 3). We applied the corrected Akaike criterion to verify whether the decay rate is better fitted by a regular Omori-like (single slope) model, or by a diffusion–Omori (double slope) model^24^, finding that the two-slope model is statistically better than the single-slope behavior, with a 55% likelihood. In the first six days after the mainshock, the slope of the line (t^−0.5^) has the characteristic decay rate of a diffusion-like phenomenon^25^, whereas after the sixth day the slope agrees with a regular Omori-like decay rate (slope ≈ 1). These observations argue for a substantial contribution of fluid pressure diffusion to the seismogenesis in the early stage (6 days) of the sequence.

### Ground displacement and seismic source model

Measured GNSS coseismic displacements well captured the cumulative coseismic deformation of the two M_w_ > 5 earthquakes, with average 1σ uncertainty of ~ 1 and 3.6 mm for the horizontal and vertical components, respectively. The largest horizontal displacements have been observed at the stations located along the coast and shows a centripetal pattern pointing toward the epicentral area (Fig. 1). The 4 closest offshore stations constrain the displacement field in the north and east zone. DARB, the closest station, and FAUZ record about 4 mm northward and about 2 mm westward, respectively. BREN and CLAW show submillimeter displacements, constraining the source length along the strike.

**Figure 4:**
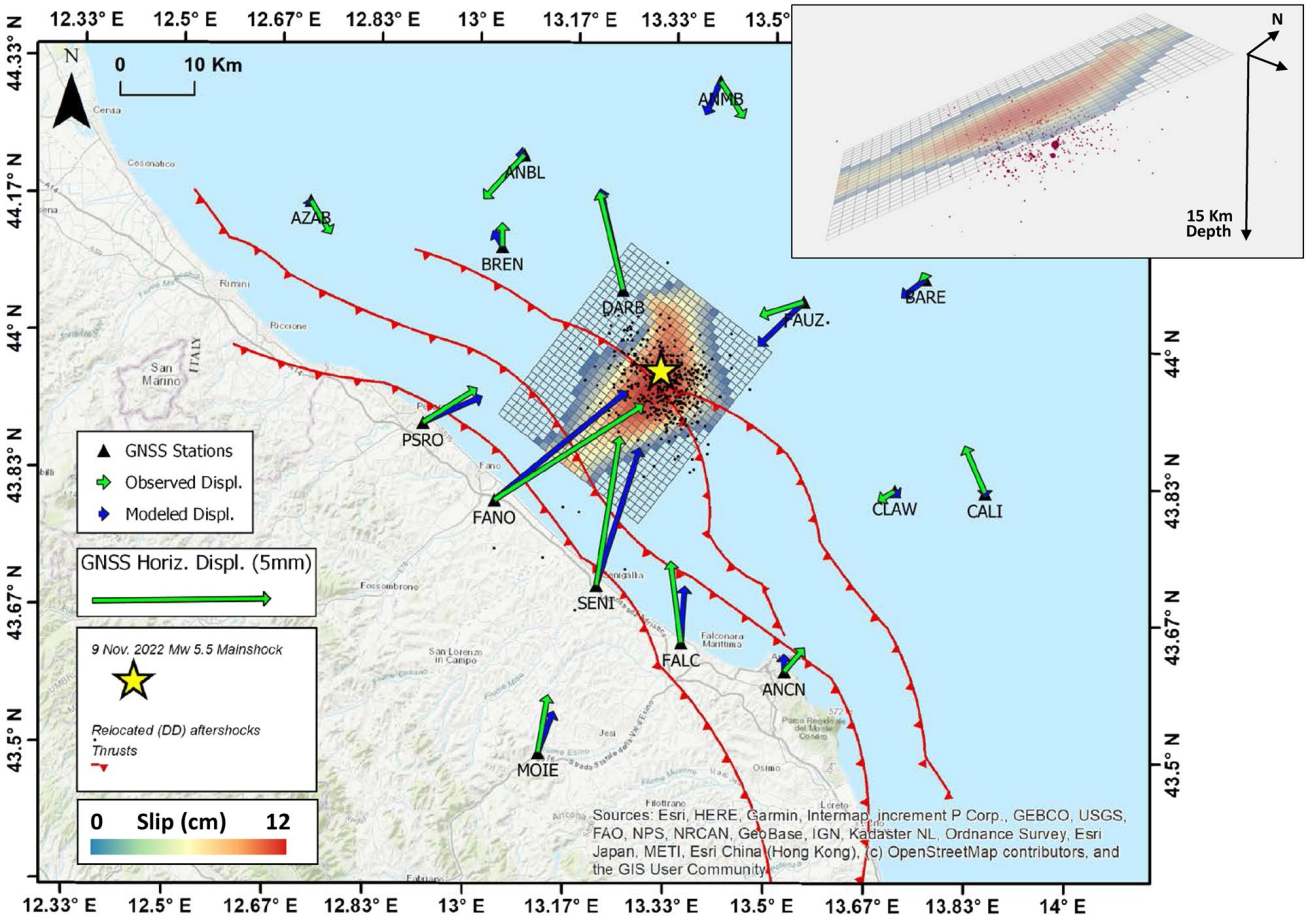
Map view of the modeled seismic source responsible for the 9 November 2022 earthquake and slip distribution along the fault, on a regular grid 1 × 1 km. Unslipped patches are displayed transparent. Black dots are the aftershocks. Green and blue arrows are the horizontal displacements recorded by the GNSS stations (black triangles) and modeled ones, respectively. We also report the main thrusts^6^. Top-Right: 3D view (from SSE) of the modeled fault plane and the DD aftershock location.

To define the seismic source responsible for the mainshock, we modeled the coseismic displacements using the Okada^26^ formalism. Geometry and kinematics of the fault are obtained in an initial unconstrained non-linear inversion of GNSS data. Best solution well reproduces the displacement pattern (Fig. S2), with a WNW-ESE striking and 24° dipping reverse fault plane, consistent with both moment tensor solution (striking 128°) and the aftershocks distribution described in the previous paragraph. Thus, we fixed to this value the strike angle of the geodetic solution. Uncertainties and trade-off parameters are shown in Fig. S3. Coseismic slip distribution along the identified fault plane is calculated by means of linear inversion. Slip along our solution concentrates between 4.5 and 10 km depth, with a maximum of about 12 cm (Fig. 4). Synthetic displacements satisfactorily fit the observed ones at the onshore stations and at on offshore ones located within a radius of 30 km from the epicenter (see also Table S1). Although noisy, the model partially reproduces the observed vector at FAUZ station. Farther ones show no displacements or values close to the uncertainty, constraining the fault dimension.

We also investigated the relationships between the mainshock source and the closest faults by means of the Coulomb Failure Function (hereinafter ΔCFF). We calculated the ΔCFF along the closest faults from DISS catalogue^5^, offshore and along the coast, caused by the 9 November earthquake. Thus, as input, we used the slip distribution estimated during the linear inversion. Stress transfer analysis reveals stress variation lower than the minimum stress-variation-threshold magnitude of 0.01 Mpa^27^ along the faults located westward of the seismic source (Fig. S4). Faults along the strike (NW and SE) of the source show values up to 0.07 MPa (to the North), and up to 0.32 Mpa along the unslipped fault portion during the 9 November earthquake.

## Discussion

The DD relocation shows that aftershocks align on a ca. 25°, SW-dipping plane between 5 and 10 km depth, although the absolute hypocentral depth of the mainshock suffers from large uncertainty. Our modeling shows a maximum slip of 12 cm on a low-angle thrust fault, whose geometry (strike and dip angles) is consistent with that indicated by aftershocks distribution, confirming the reliability of our modeling results. The coseismic slip mainly concentrates at depth from the mainshock hypocenter, suggesting a downward and coast-ward directivity of the coseismic rupture.

The sequence has a magnitude distribution of events with a b-value close to 1 and shows a first diffusive pattern (0.5 slope in Fig. 2d) followed by a normal decay (slope 1), with an oscillating release of energy (Fig. 2c) that tends to be Omori-like. The aftershocks diffusion is usually interpreted as a diffusion of the stress induced by the mainshock, either by fluid transfer in the crust^25,28^, or due to a viscous relaxation process^29^. Indeed, the seismicity diffusion may also result from a simple cascade process where the mainshock triggers aftershocks that in turn trigger their own aftershocks, leading thus to a general expansion of the aftershock volume. We hypothesize that after an external forcing that locally increased the seismicity rate during the very first part of the sequence, diffusion was reduced leading to a standard decay. This inferred Omori trend is like that of the Emilia 2012 seismic sequence^30^, but the initial diffusive pattern was limited to the first 4–5 days and did not lead to the occurrence of further mainshocks. Trying to estimate in semi real time such patterns could be relevant to make inference on the possible evolution of the sequence, but caution should be exercised when using data of an ongoing seismic sequence^31^.

The compressional structure (fault-related fold) is well illuminated in public seismic reflection profiles but the definition of the activated thrust portion is hindered by the high noise versus signal ratio affecting the seismic data at depths larger than 2 s TWT (Fig. 2b). Shallow buried anticlines are well mapped in the upper 5–6 crustal kilometers^20–22^. They developed above 20°–35° dipping ramps splaying from a regional basal décollement located at depth and dipping between 1° and 7° toward the west^32^. One of the above-mentioned ramps seems to be the fault plane activated during the earthquake and it might correspond to the NAF-10 structure^21^. Similarly to the 20 May 2012 Mw6.0 Emilia and the 26 November 2019 Mw6.4 Durrës compressional earthquakes, also in this case the coseismic slip propagated from the hypocenter downward^30,33,34^, a feature typical of thrust faults^35^. This suggests that a crucial role in the future evolution of the sequence could be played by the stress increase (up to 0.31 Mpa) along the un-slipped portions of the activated thrust, and, more in general, along the thrust-front. Under this assumption, we expect to observe an along-strike progression of the sequence with minor impacts on the coastal belt.

## Conclusions

The analysis of geodetic and seismological data available since a few days after the 9 November 2022 earthquake in the northern Adriatic area defines a set of constraints about the seismic sequence and criticalities in the source determination, as well as the crucial role of geophysical/geodetic data acquired by hydrocarbon industry infrastructures. Monitoring of buried faults in offshore areas can take great advantage from the presence of industrial infrastructures that could host GNSS continuous stations. This aspect, coupled with amphibious seismic networks are indispensable for monitoring such faults and constrain seismological parameters.

Hypocenters distribution and GNSS coseismic displacements inversion reveal that slip occurred along a 15 km long fault patch along a ca. 24° SSW-dipping thrust fault, consistent with seismic reflection data propagating downward from the mainshock hypocenter. Slip amount rapidly decreased upward and along strike, where stress accumulated. If, on one hand, the downward pattern of slip intuitively reduces the tsunamigenic potential, on the other hand the stress increase bordering the narrow slip distribution holds a pointed light on the sequence evolution.

The 2022 Adriatic compressional earthquake sequence confirms the ongoing seismotectonic activity of this sector of the Apennines that is still propagating toward its foreland roughly in a piggy-back thrust sequence.

## Methods

We relocated 594 aftershocks in the period 22 November 2022–05 January 2023 and the 09 November mainshock by using the hypoDD relative method^8^. We used the *P-* and *S-*wave arrival times picked by the seismologists on duty in the INGV control room, obtaining 30,226 P-wave and 12,739 S-wave differential times computed from the initial P and S wave phase readings at 55 stations. We optimized the damping parameter and verified the convergence of the solution with a final rms of 0.13 s. The accuracy of the hypocenter solutions has been estimated by using a bootstrap approach. Starting from the residual vector derived from the double difference inversion, we use the bootstrap resampling method^36^ to create 200 random residual vectors of unit weight that we add to the observed differential travel times. This results in 200 bootstrap datasets that are inverted as in the real case. For each event we thus obtain 200 values for the hypocenter parameters and this allows us to infer a realistic estimation of the uncertainties, that are within 100 m on average.

We report on the histograms of Fig. S5 of the supplementary material the average value –computed on 200 bootstrap samples– of the uncertainties on the X, Y, and Z coordinates. The mainshock relocation is obtained by using the hypoDD method after a re-picking of the P- and S-phase. In order to verify if that the hypoDD relocation does not cause a significant shift of the entire earthquakes cluster (a circumstance that may raise from the relative relocation method), we compare the hypoDD relocation shown in Fig. 3a, b with the 1D location. The result of this exercise is shown into a new Figure (Fig. S6) of the supplementary material.

To characterize the seismic sequence in terms of magnitude distribution and decay we have analyzed data from the seismic control room. By using as input the earthquake relocated catalog and by adopting a maximum likelihood estimation approach^37^, we estimated the magnitude distribution of the seismic sequence following the Gutenberg-Richter^23^ recurrence relation and adopting:

$$logN\left(M\right)=a-bM$$

where *a* and *b* represent the annual level of seismicity and the ratio between the number of small and large earthquakes, respectively; *N*(*M*) is the cumulative number of earthquakes with magnitude *M* and larger within the used catalog.

Aftershocks/day and the daily average energy release is plotted as a function of time. The completeness magnitude threshold of 2.0 which is reached 4 h after the mainshock. Uncertainties on the rates were calculated by bootstrapping the data sets with 100 realizations and indicated with vertical bars at each data point in Fig. 3.

Raw daily GNSS observations were processed by using the GAMIT/GLOBK software^9,38^ to estimate the coseismic displacements (additional details in supplementary material). The analyzed dataset includes 15 GNSS stations (Table 1) located within 60 km from the epicenter, from 1 October to 10 November 2022. Most of these stations are property of Eni S.p.A. and are located on onshore and offshore industrial infrastructures (e.g., seabed-anchored platforms and storage centers); the other stations belong to the NetGEO network (http://www.netgeo.it), owned by Topcon positioning and INGV RING network (http://ring.gm.ingv.it ). To improve the overall configuration of the network and tie the regional measurements to an external global reference frame, data coming from 15 continuous stations belonging to EUREF (https://epncb.oma.be), ASI (http://geodaf.mt.asi.it) and FReDNet (https://frednet.crs.ogs.it/DOI/) were introduced in the processing. The daily estimates of loosely constrained station coordinates have been combined in GLOBK to estimate a consistent set of daily coordinates (i.e., time-series) for all processed stations. Some stations show a significant offset on their time-series, therefore we computed the amount of 3D coseismic displacements, by differencing the average sites position in the three days before and the two days after the two main events with minimal constraints (i.e., constraining translations, scale, and rotations of the network solution to 0.1 mm). Due to the very short time between the occurrence of the two M_w_ > 5 earthquakes, the estimated displacement pattern represents the cumulative effects of both shocks. Uncertainties associated with the coseismic displacements have been estimated as the “sum in quadrature”^39^ of the errors associated with the average sites position before and after the events. As mentioned above, average uncertainty for the horizontal and vertical components are ~ 1 and 3.6 mm, respectively. These estimations must be considered as an overestimation of the real ones, due to the conservative approach used to their computation.

**Table 1:** Coseismic displacement values; 1-sigma uncertainties are also reported.

Site_id	Long (°)	Lat (°)	ΔE (mm)	ΔN (mm)	ΔU (mm)	σΔE (mm)	σΔN (mm)	σΔU (mm)
ANBL*	13.0788	44.2285	− 1.2	− 1.3	5.2	1.0	1.1	3.6
ANCN	13.5316	43.6072	0.6	0.7	2.1	0.5	0.5	1.7
ANMB*	13.4072	44.3226	0.7	− 1.1	− 0.3	1.5	1.7	5.6
AZAB*	12.7205	44.1667	0.6	− 1.0	0.5	0.8	1.0	3.1
BARE*	13.7579	44.0863	− 0.1	0.2	− 2.3	1.3	1.4	4.5
BREN*	13.0450	44.1164	0.0	0.7	1.4	0.9	1.0	3.2
CALI*	13.8634	43.8273	− 0.6	1.4	2.2	0.9	0.9	3.1
CLAW*	13.7118	43.8285	− 0.5	− 0.3	0.6	0.9	0.9	3.1
DARB*	13.2497	44.0670	− 0.7	2.9	1.9	1.2	1.3	4.3
FALC*	13.3583	43.6401	− 0.3	2.4	1.5	0.9	1.0	3.6
FANO*	13.0412	43.8086	4.4	2.8	2.9	1.0	1.1	3.7
FAUZ*	13.5540	44.0564	− 1.3	− 0.4	− 2.1	1.6	1.8	5.6
MOIE	13.1235	43.5032	0.3	1.7	4.5	1.0	1.1	3.6
PSRO	12.9184	43.9007	1.6	1.0	2.1	0.7	0.9	2.8
SENI	13.2150	43.7076	0.7	4.4	4.6	0.6	0.6	2.1

*Eni S.p.A. GNSS stations.

Seismic source model is performed using the Okada formalism^26^, implemented in the SARscape software by SARmap (sarmap SA, 5.6 version), working in the ENVI® (L3HARRIS) environment. First, we performed an unconstrained non-linear inversion of the coseismic displacements measured by GNSS stations, to retrieve geometry and kinematics of the fault, followed by a linear inversion to estimate the slip distribution along the plane^40^. Because of the large uncertainties, the contribution of the vertical component in the uncertainty-weighted inversion is negligible, we considered the only horizontal vectors in the inversion. In supplementary materials we show the slip uncertainty (1σ predicted slip error standard deviation) along the fault plane (Fig. S7). To visualize the slip resolution in depth, we performed an additional linear model to retrieve the slip distribution along the fault plane using a variable size grid (Figs. S8 and S9). In our approach^41^ patch dimensions are automatically determined by the inversion algorithm maximizing the model resolution matrix^42^. Patches vary from 3 × 4 km at shallow depths to 8 × 6 km at 15 km depth. Following the same approach we estimated uncertainties^40^.

The relationships between the mainshock source and the closest faults is investigated by using the Coulomb Failure Function. Results of seismic source modeling, seismological analysis and subsequent interpretation reveal the activation of a low angle thrust, corresponding to the ITCS106—Pesaro mare—Cornelia Composite Source, from DISS catalogue. Thus, we refined and tune the DISS source, adapting to our modelling result to calculate the stress increase along the activated fault plane. The underground fluid pressure is not considered and the friction coefficient is considered constant at 0.4. Being the absolute stress value unknown, the ΔCFF is defined as^43^:

$$\Delta CFF=\Delta \tau +\mu {\prime}\Delta \sigma$$

in which τ is the shear traction, σ is the normal traction (positive for traction) and *μ’* is the apparent friction coefficient. Stress change is computed assuming that the half-space is a Poisson solid (ν = 0.25) with Young modulus (E) of 75 GPa and a shear modulus (G) of 30 GPa.

## Supplementary Information

Supplementary Information.

## Data Availability

GNSS solutions are reported in Table 1 whereas raw data are available INGV RING network web site (http://ring.gm.ingv.it; ftp://bancadati2.gm.ingv.it:2121/OUTGOING/RINEX30/RING/). Reflection seismic data have been supplied by Ministry for Economic Development and available at http://www.videpi.com; https://www.arcgis.com/home/webmap/viewer.html?webmap=7596b9556827437b8b540d52bf3dbff4&extent=9.6927,41.1093,17.8061,44.7086. Seismological raw data are accessible at http://terremoti.ingv.it/ and https://eida.ingv.it/en/.

## References

[1] Pezzo G (2020). Active Fold-thrust belt to foreland transition in Northern Adria, Italy, tracked by seismic reflection profiles and GPS offshore data. Tectonics.

[2] Serpelloni E, Cavaliere A, Martelli L, Pintori F, Anderlini L, Borghi A, Randazzo D, Bruni S, Devoti R, Perfetti P, Cacciaguerra S (2022). Surface velocities and strain-rates in the Euro-Mediterranean region from massive GPS data processing. Front. Earth Sci..

[3] Di Bucci D, Mazzoli S (2002). Active tectonics of the Northern Apennines and Adria geodynamics: New data and a discussion. J. Geodyn..

[4] Trippetta F (2019). From mapped faults to fault-length earthquake magnitude (FLEM): A test on Italy with methodological implications. Solid Earth.

[5] DISS Working Group (2021). Database of individual seismogenic sources (DISS), version 3.3.0: A compilation of potential sources for earthquakes larger than M 5.5 in Italy and surrounding areas.

[6] Fantoni R, Franciosi R (2010). Tectono-sedimentary setting of the Po Plain and Adriatic foreland. Rendiconti Lincei..

[7] Scognamiglio L, Tinti E, Michelini A (2009). Real-time determination of seismic moment tensor for the Italian region. Bull. Seismol. Soc. Am..

[8] Waldhauser F, Ellsworth WL (2000). A double-difference earthquake location algorithm: Method and application to the northern Hayward fault, California. Bull. Seismol. Soc. Am..

[9] Palano M (2020). Geopositioning time series from offshore platforms in the Adriatic Sea. Sci. Data.

[10] Haines B, Desai SD, Kubitschek D, Leben RR (2021). A brief history of the Harvest experiment: 1989–2019. Adv. Space Res..

[11] Carminati E, Lustrino M, Doglioni C (2012). Geodynamic evolution of the central and western Mediterranean: Tectonics vs. igneous petrology constraints. Tectonophysics.

[12] Bally AW (1986). Balanced sections and seismic reflection profiles across the Central Apennines. Mem. Soc. Geol. It..

[13] Argnani A (1998). Structural elements of the Adriatic foreland and their relationships with the front of the Apennine fold-and-thrust belt. Mem. Soc. Geol. Ital..

[14] Del Ben A (2002). Interpretation of the CROP M-16 seismic section in the Central Adriatic Sea. Mem. Della Soc. Geol. Ital..

[15] Maesano FE, Toscani G, Burrato P, Mirabella F, D'Ambrogi C, Basili R (2013). Deriving thrust fault slip rates from geological modeling: Examples from the Marche coastal and offshore contraction belt, Northern Apennines, Italy. Mar. Pet. Geol..

[16] Barchi MR, De Feyter A, Magnani MB, Minelli G, Pialli G, Sotera BM (1998). The structural style of the Umbria-Marche fold and thrust belt. Mem. Della Soc. Geol. Ital..

[17] Kastelic V, Vannoli P, Burrato P, Fracassi U, Tiberti MM, Valensise G (2013). Seismogenic sources in the Adriatic Domain. Mar. Pet. Geol..

[18] Bennett RA, Serpelloni E, Hreinsdóttir S, Brandon MT, Buble G, Basic T, Casale G, Cavaliere A, Anzidei M, Marjonovic M, Minelli G, Molli G, Montanari A (2012). Syn-convergent extension observed using the RETREAT GPS network, northern Apennines, Italy. J. Geophys. Res. Solid Earth.

[19] Devoti R, d’Agostino N, Serpelloni E, Pietrantonio G, Riguzzi F, Avallone A, Cavaliere A, Cheloni D, Cecere G, D'Ambrosio C, Falco L, Selvaggi G, Métois M, Esposito A, Sepe V, Galvani A, Anzidei M (2017). A combined velocity field of the Mediterranean region. Ann. Geophys.

[20] Casero P, Bigi S (2013). Structural setting of the Adriatic basin and the main related petroleum exploration plays. Mar. Pet. Geol..

[21] Panara Y, Maesano FE, Amadori C, Fedorik J, Toscani G, Basili R (2021). Probabilistic assessment of slip rates and their variability over time of offshore buried thrusts: A case study in the Northern Adriatic Sea. Front. Earth Sci..

[22] Maesano FE (2023). Buried alive: Imaging the 9 November 2022, M_w_ 5.5 earthquake source on the offshore Adriatic blind thrust front of the Northern Apennines (Italy). Geophys. Res. Lett..

[23] Gutenberg B, Richter CF (1944). Frequency of earthquakes in California. Bull. Seismol. Soc. Am..

[24] Malagnini L, Lucente FP, De Gori P, Akinci A, Munafò I (2012). Control of pore fluid pressure diffusion on fault failure mode: Insights from the 2009 L’Aquila seismic sequence. J. Geophys. Res. Solid Earth.

[25] Nur A, Booker JR (1972). Aftershocks caused by pore fluid flow?. Science.

[26] Okada Y (1985). Surface deformation due to shear and tensile faults in a half-space. Bull. Seismol. Soc. Am..

[27] Hardebeck JL, Nazareth JJ, Hauksson E (1998). The static stress change triggering model: Constraints from two southern California aftershock sequences. J. Geophys. Res. Solid Earth.

[28] Hudnut KW, Seeber L, Pacheco J (1989). Cross-fault triggering in the November 1987 superstition hills earthquake sequence, southern California. Geophys. Res. Lett..

[29] Rydelek PA, Sacks IS (2001). Migration of large earthquakes along the San Jacinto fault; Stress diffusion from the 1857 Fort Tejon earthquake. Geophys. Res. Lett..

[30] Pezzo G, De Gori P, Lucente FP, Chiarabba C (2018). Pore pressure pulse drove the 2012 Emilia (Italy) series of earthquakes. Geophys. Res. Lett..

[31] Spassiani I, Taroni M, Murru M, Falcone G (2023). Real time Gutenberg-Richter b-value estimation for an ongoing seismic sequence: An application to the 2022 marche offshore earthquake sequence (ML 5.7 central Italy). Geophys. J. Int..

[32] Mariotti G, Doglioni C (2000). The dip of the foreland monocline in the Alps and Apennines. Earth Planet. Sci. Lett..

[33] Pezzo G, Palano M, Chiarabba C (2022). Rotation at subduction margins: How complexity at fault-scale (the 2019 Albanian Mw 6.4 earthquake) mirrors the regional deformation. Terra Nova.

[34] Teloni S, Invernizzi C, Mazzoli S, Pierantoni PP, Spina V (2021). Seismogenic fault system of the Mw 6.4 November 2019 Albania earthquake: New insights into the structural architecture and active tectonic setting of the outer Albanides. J. Geol. Soc..

[35] Carminati E, Doglioni C, Barba S (2004). Reverse migration of seismicity on thrusts and normal faults. Earth Sci. Rev..

[36] Efron B (1982). The Jackknife, the Bootstrap, and Other Resampling Plans.

[37] Aki K (1965). Maximum likelihood estimate of *b* in the formula log *N* = *a – Bm* and its confidence limits. Bull. Earthq. Res. Inst..

[38] Herring TA, King RW, Floyd MA, McClusky SC (2018). Introduction to GAMIT/GLOBK, Release 10.7.

[39] Taylor JR (1997). An Introduction to Error Analysis: The Study of Uncertainties in Physical Measurements.

[40] Atzori S, Manunta M, Fornaro G, Ganas A, Salvi S (2008). Postseismic displacement of the 1999 Athens earthquake retrieved by the differential interferometry by synthetic aperture radar time series. J. Geophys. Res. Solid Earth.

[41] Atzori S, Antonioli A (2011). Optimal fault resolution in geodetic inversion of coseismic data. Geophys. J. Int..

[42] Menke W (1989). Geophysical Data Analysis: Discrete Inverse Theory.

[43] Harris RA (1998). Introduction to special section: Stress triggers, stress shadows, and implications for seismic hazard. J. Geophys. Res. Solid Earth.

[44] Kanamori H, Mori J, Hauksson E, Heaton TH, Hutton K, Jones LM (1993). Determination of earthquake energy release and ml using Terrascope. Bull. Seismol. Soc. Am..

