# Supplementary material for: Seismic source identification of the 9 November 2022 Mw 5.5 offshore Adriatic sea (Italy) earthquake from GNSS data and aftershock relocation
Source: Sci Rep. 2023 Jul 16;13:11474. doi: 10.1038/s41598-023-38150-5 (PMC10350452; doi:10.1038/s41598-023-38150-5)
Supplement: Supplementary file 1 — Supplementary Information. [file 41598_2023_38150_MOESM1_ESM.docx]

Supporting Information for

**Seismic source identification of the 9 November 2022 Mw 5.5 offshore Adriatic sea (Italy) earthquake from GNSS data and aftershock relocation.**

Pezzo G. ^1^ *, Billi, A.^2^, Carminati, E. ^3^, Conti, A. ^2^, De Gori, P. ^1^, Devoti, R. ^1^, Lucente, F.P. ^1^, Palano, M. ^4,2^, Petracchini. L. ^2^, Serpelloni, E. ^5^, Tavani, S., ^6,2^ and Chiarabba, C^1^.

^1^ Istituto Nazionale di Geofisica e Vulcanologia, Osservatorio Nazionale Terremoti, Rome, Italy

^2^ Consiglio Nazionale delle Ricerche, IGAG, Rome, Italy

^3^ Dip. Scienze della Terra, Sapienza Università di Roma, Rome, Italy,

^4^ Istituto Nazionale di Geofisica e Vulcanologia, Osservatorio Etneo, Catania, Italy

^5^ Istituto Nazionale di Geofisica e Vulcanologia, Sezione di Bologna, Bologna, Italy

^6^ DISTAR, Università degli Studi di Napoli “Federico II,” Via Cupa Nuova Cintia 21, 80126, Naples, Italy

*Corresponding author

**Contents of this file**

Text S1

Figures S1 to S9

Table S1

References

**Introduction**

We provide additional information related to geodetic data processing along with some additional figures.

**Text S1.**

Due to the offshore location of both mainshocks we get GNSS data from Eni S.p.A., an Oil&Gas operator which in the framework of the hydrocarbon exploitation is also monitoring the coastal and seafloor deformation with GNSS stations installed on industrial infrastructures as seabed-anchored platforms and onshore storage centers [1]. These data, along with the other stations (mainly from TopCon Netgeo network; <http://shop.netgeo.it/> ) located in a 60 km radius from the epicenter and spanning the 1 October - 10 November 2022 time interval, were processed by using the GAMIT/GLOBK software [2] following the strategy described in Palano et al. (2020) [1]. To improve the overall configuration of the network and tie the regional measurements to an external global reference frame, data coming from 15 continuous stations belonging to EUREF (<https://epncb.oma.be>), ASI (<http://geodaf.mt.asi.it>) and FReDNet (<https://frednet.crs.ogs.it/DOI/>) were introduced in the processing. The daily estimates of loosely constrained station coordinates have been combined in GLOBK estimate a consistent set of daily coordinates (i.e., time-series) for all processed stations. Some stations show a significant offset on their time-series, therefore we computed the amount of 3D coseismic displacement events, by differencing the average sites position in the three days before and the two days after the two main events with minimal constraints (i.e., constraining translations, scale and rotations of the network solution to 0.1 mm). Achieved displacement field is reported in Figure 2.


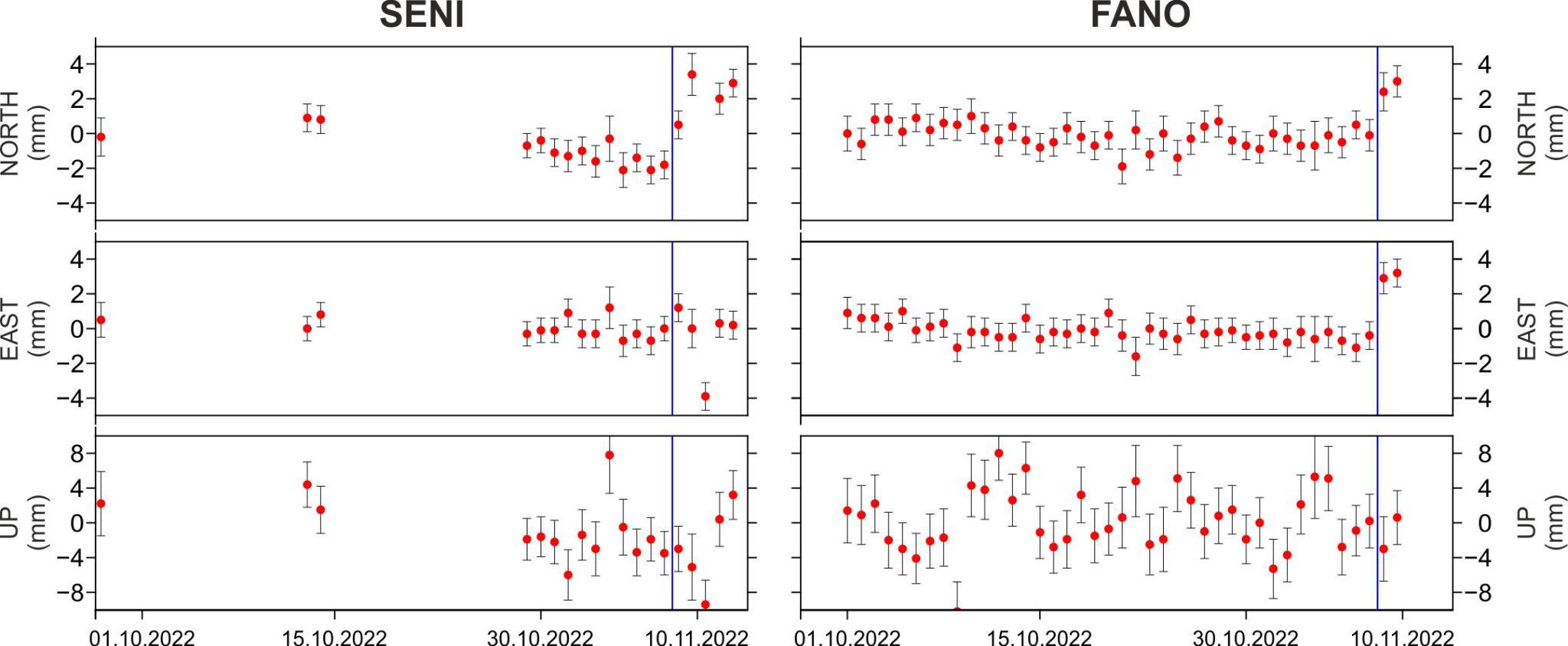


**Figure S1.** Time-series of SENI and FANO GNSS stations. The vertical blue line stands for the M5.5 earthquake.

**
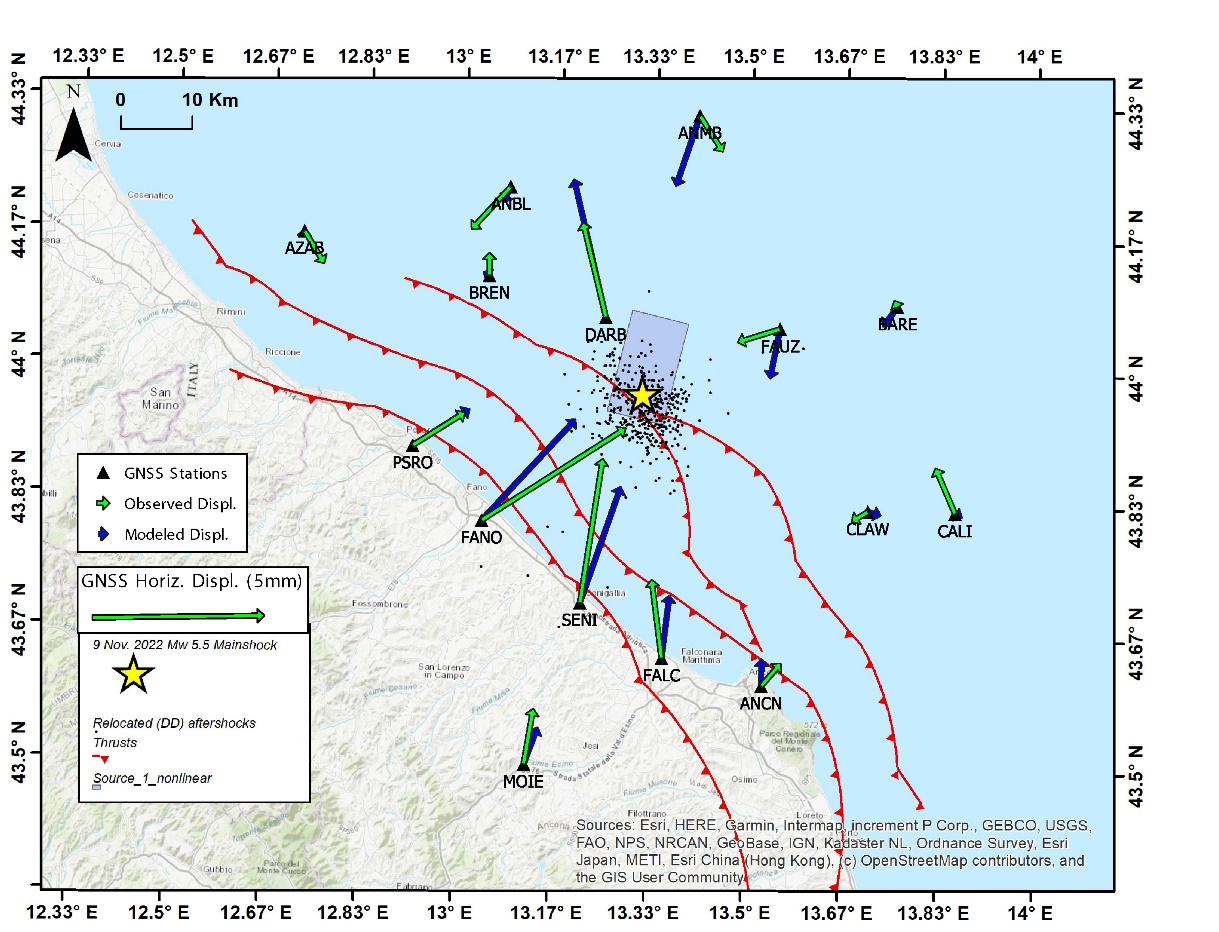
**

**Figure S2.** Non-linear-inversion result. Map view of the modeled seismic source responsible for the 9 November 2022 earthquake. Black dots indicate the aftershocks. Green and red arrows are the coseismic displacements recorded by the GNSS stations and modeled ones, respectively. In the map we report the surface projections of the main thrust close to the seismic sequence [3].


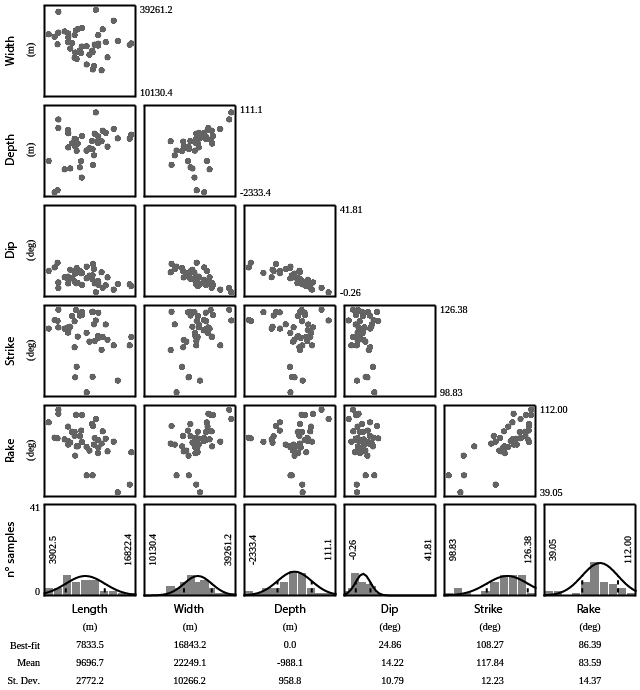


**Figure S3.** Parameter uncertainties, best fit and trade-offs estimated with 150 restarts of the inversion, adding, each time, a synthetic noise [4].


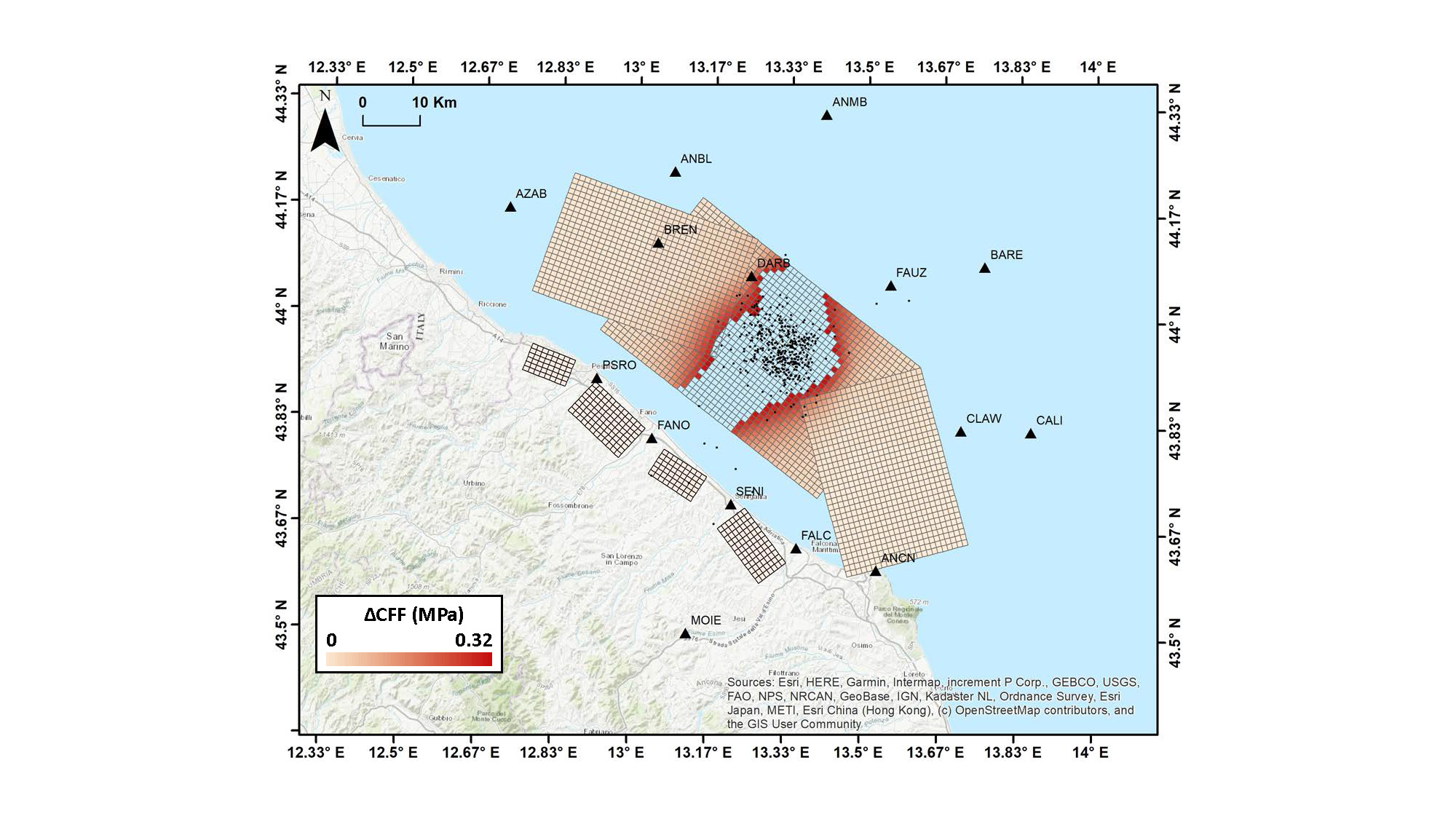


**Figure S4.** Static stress variation due to the 9 November 2022 earthquake calculated along closest fault planes located offshore and along the coast (as defined by Diss Working Group, 2021) [5].


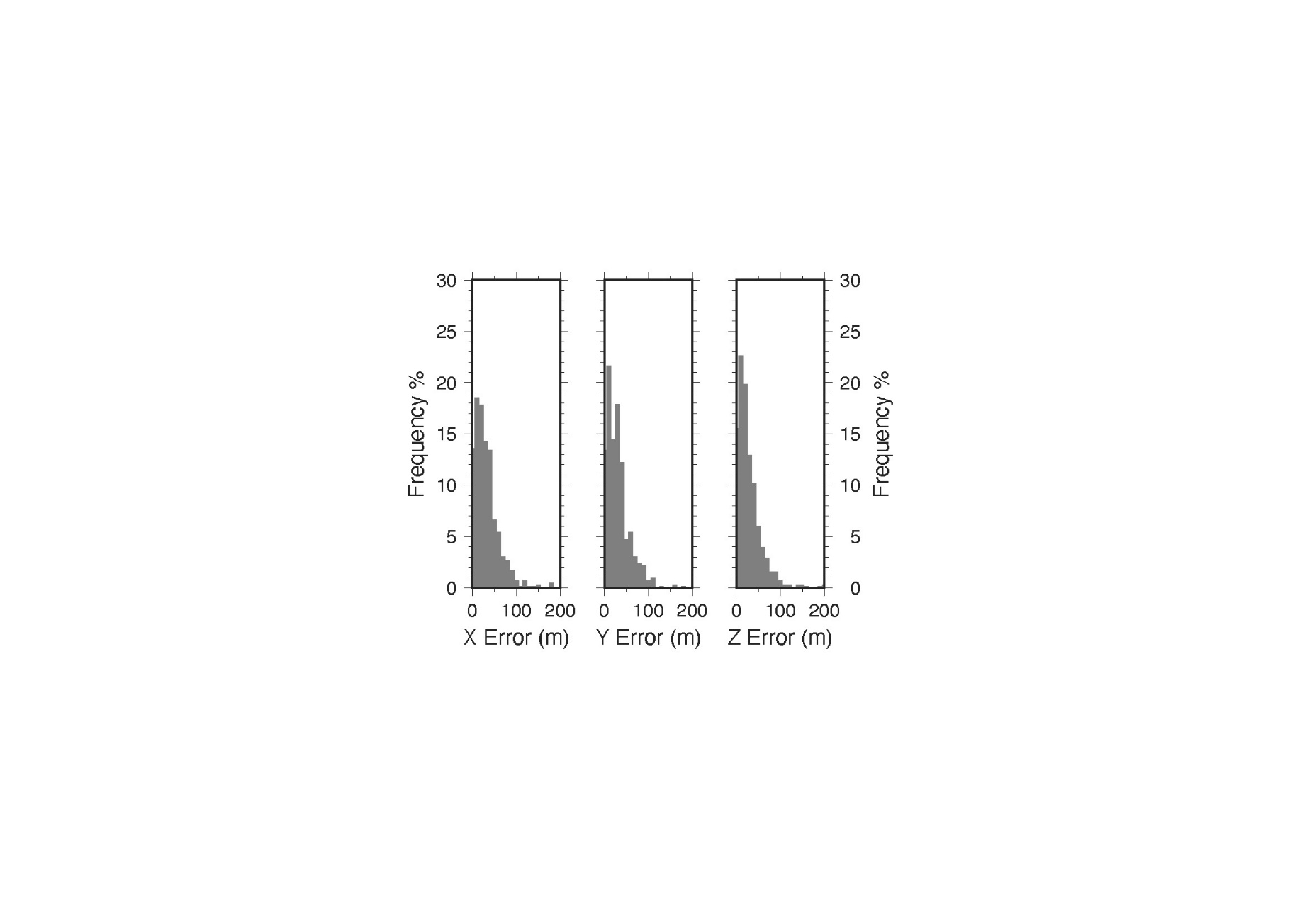


**Figure S5**. Histograms showing the frequency distribution of the average value –computed on 200 bootstrap samples– of the uncertainties on the X, Y, and Z coordinates of the hypocenter localizations.


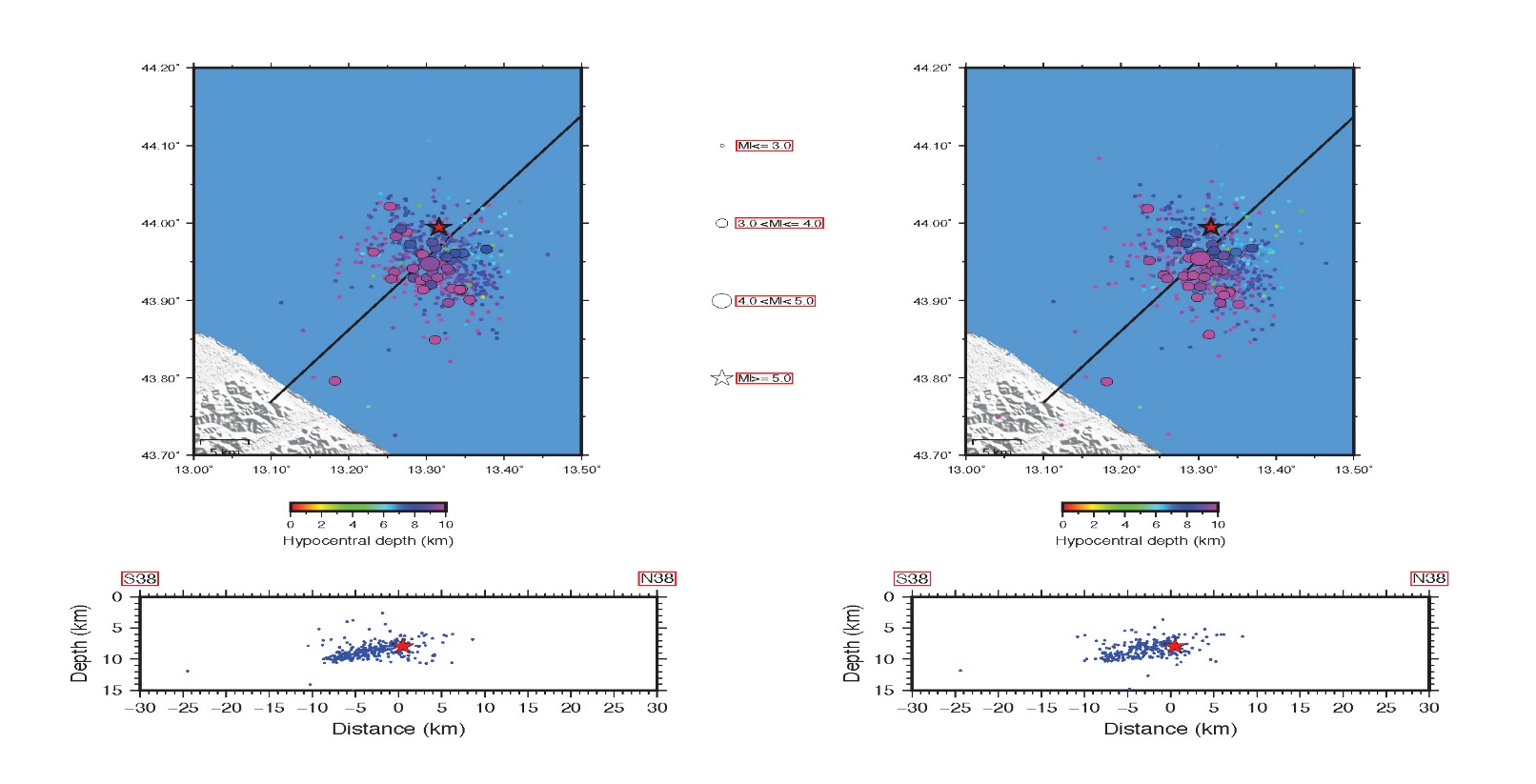


**Figure S6**. Comparison between the hypoDD (on the left) and 1D (on the right locations, both in maps (above panels) and in cross section (below panels). The trace of the cross section –shown on the maps– is the same as Figure 3b of the main text. Earthquakes are projected within 3 km from the cross section vertical plane. Red star on each panel represents the mainshock hypocenter.


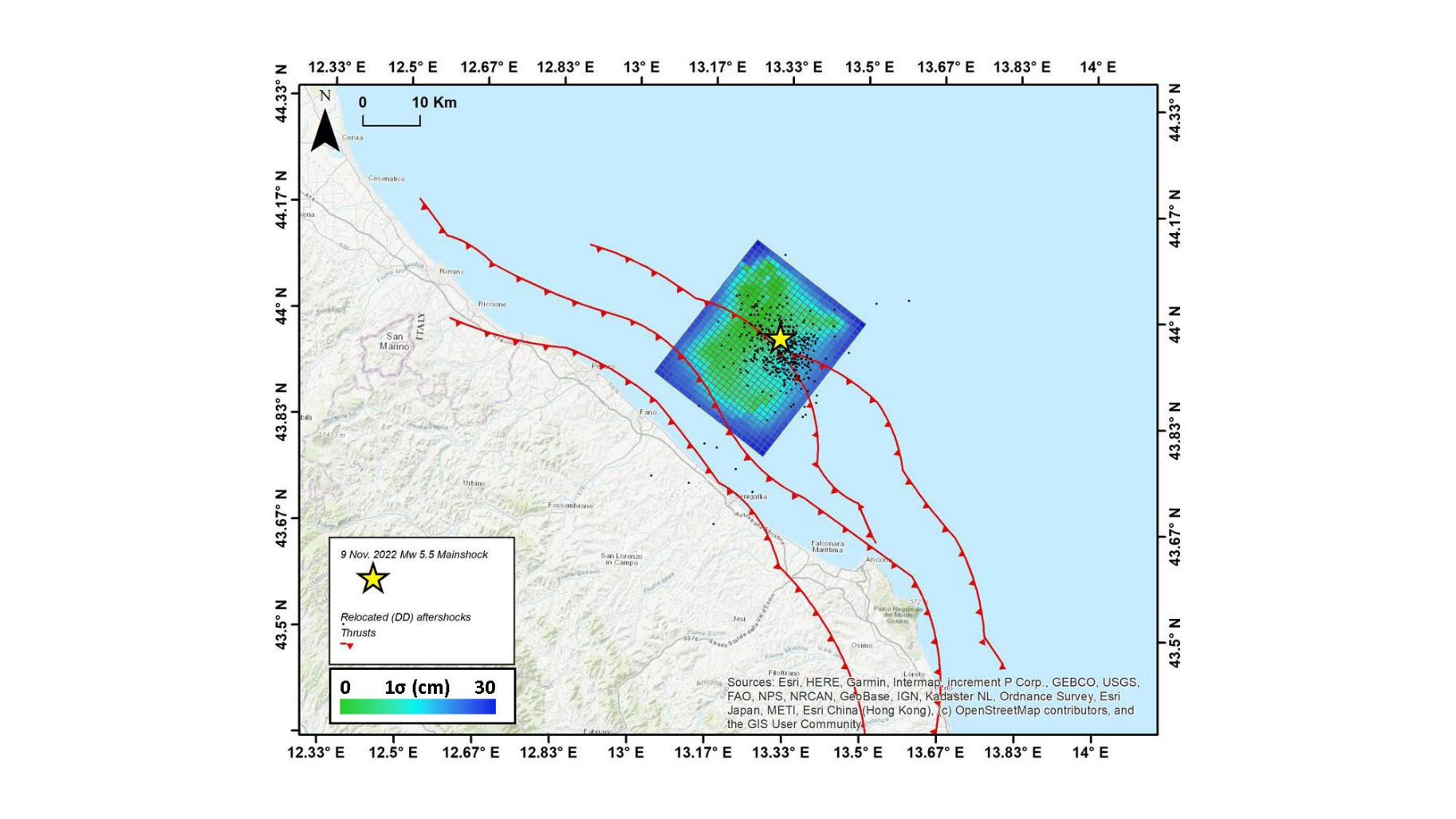


**Figure S7.** Slip uncertainty (1σ predicted slip error standard deviation) along the fault plane calculated during the linear inversion (map view). Black dots are the relocated aftershocks and yellow star is the Mw 5.5 mainshock. In the map we report the surface projections of the main thrusts [3].


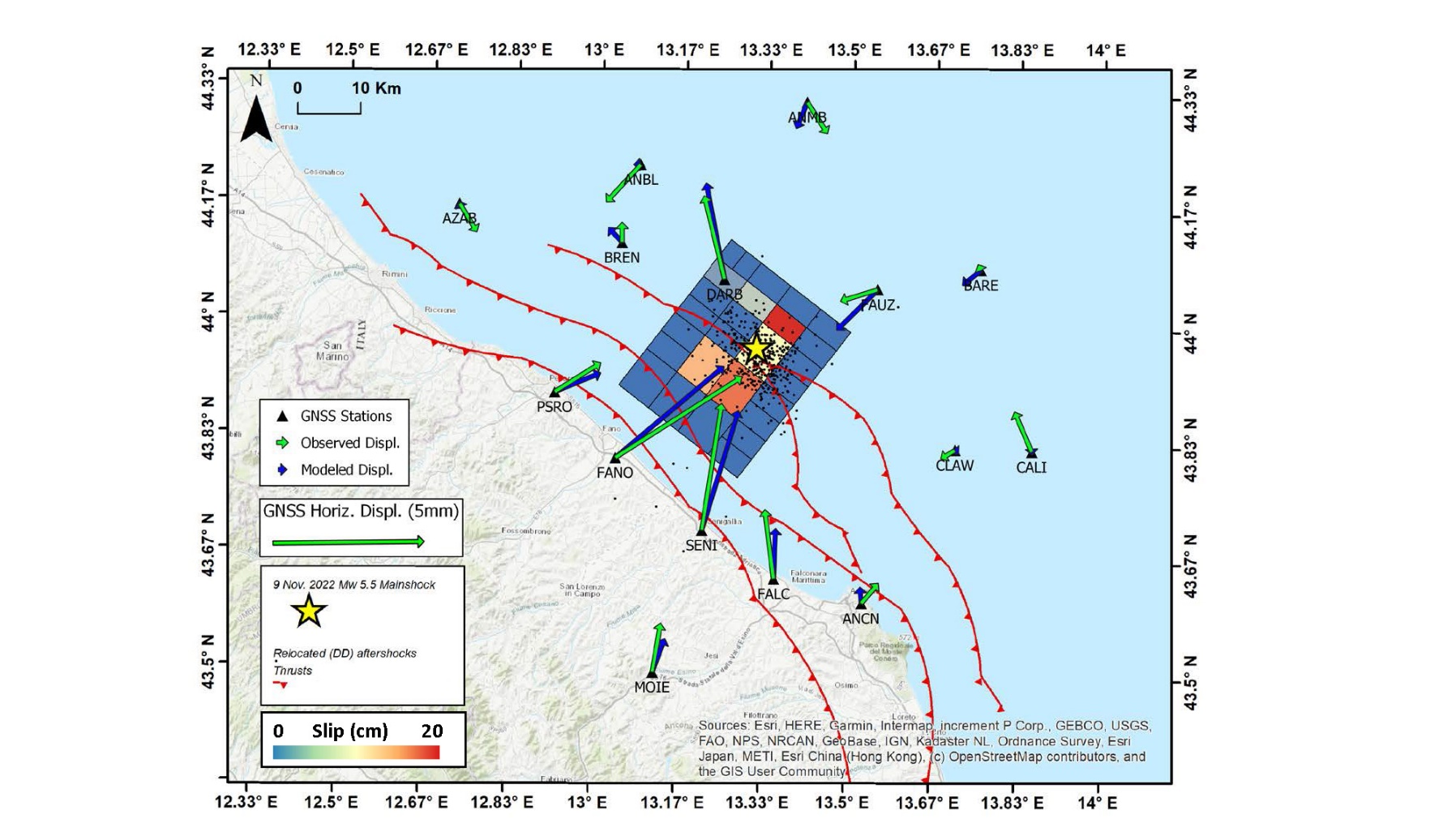


**Figure S8.** Variable patch model results. Map view of the modeled seismic source responsible for the 9 November 2022 earthquake calculated using a variable grid-size of the modeled fault plane. Green and blue arrows are the coseismic displacements recorded by the GNSS stations (black triangles) and modeled ones, respectively. Black dots are the relocated aftershocks and yellow star is the Mw 5.5 mainshock. In the map we report the surface projections of the main thrusts [3].


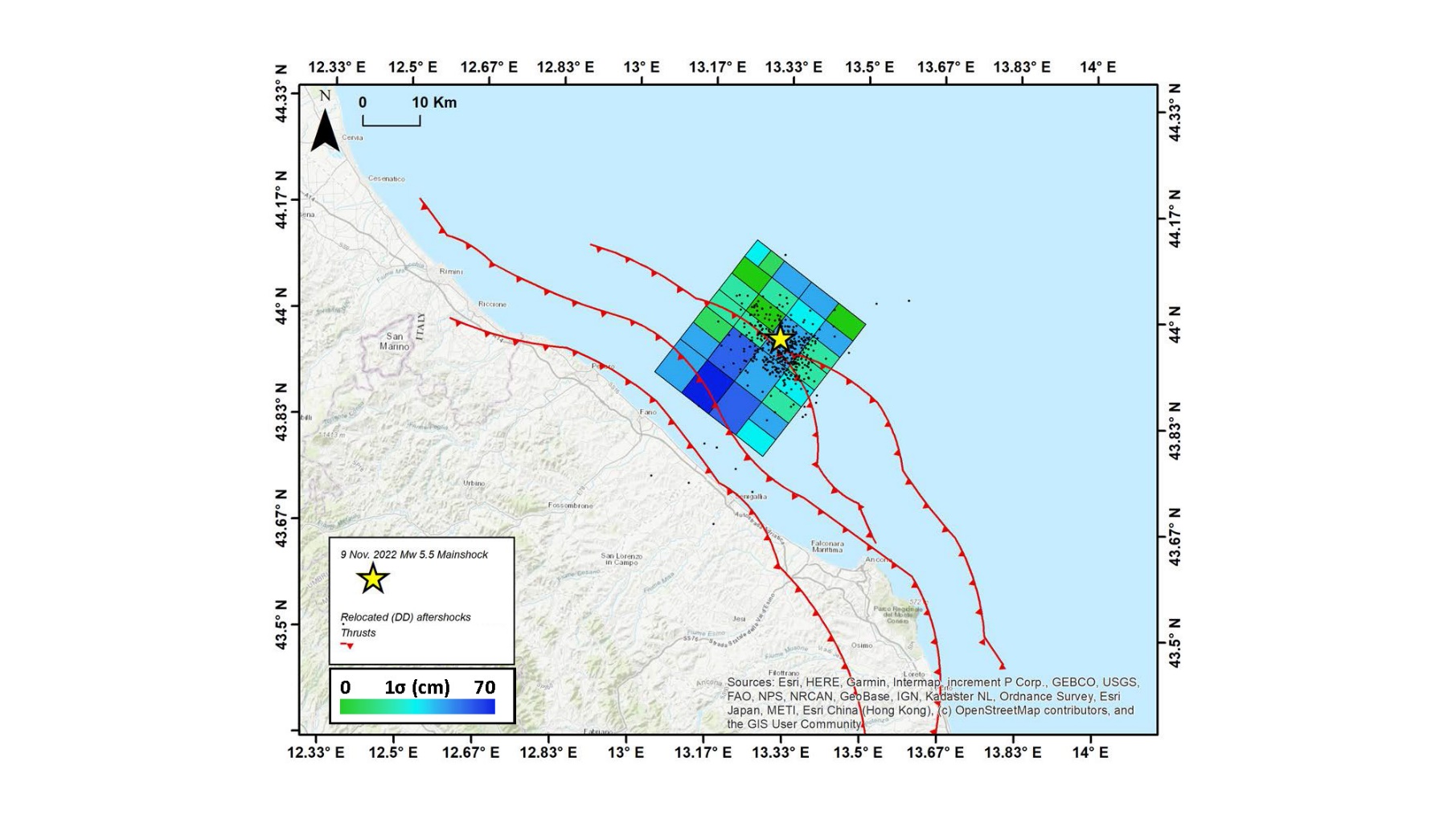


**Figure S9.** Slip uncertainty (1σ predicted slip error standard deviation) along the fault plane calculated during the variable-size-patch linear inversion (map view). Black dots are the relocated aftershocks and yellow star is the Mw 5.5 mainshock. In the map we report the surface projections of the main thrusts [3].

**Tables**

| **Site_id** | **Long** (°) | **Lat** (°) | **Observed**  **ΔE** (mm) | **Modeled**  **ΔE** (mm) | **Observed**  **ΔN** (mm) | **Modeled**  **ΔN** (mm) |
| --- | --- | --- | --- | --- | --- | --- |
| ANBL* | 13.0788 | 44.2285 | -1.2 | -0.3 | -1.3 | 0.0 |
| ANCN | 13.5316 | 43.6072 | 0.6 | 0.0 | 0.7 | 0.5 |
| ANMB* | 13.4072 | 44.3226 | 0.7 | -0.5 | -1.1 | -1.0 |
| AZAB* | 12.7205 | 44.1667 | 0.6 | 0.0 | -1.0 | 0.1 |
| BARE* | 13.7579 | 44.0863 | -0.1 | -0.7 | 0.2 | -0.5 |
| BREN* | 13.0450 | 44.1164 | 0.0 | -0.3 | 0.7 | 0.5 |
| CALI* | 13.8634 | 43.8273 | -0.6 | 0.0 | 1.4 | -0.1 |
| CLAW* | 13.7118 | 43.8285 | -0.5 | 0.2 | -0.3 | -0.2 |
| DARB* | 13.2497 | 44.0670 | -0.7 | -0.7 | 2.9 | 3.0 |
| FALC* | 13.3583 | 43.6401 | -0.3 | 0.1 | 2.4 | 1.7 |
| FANO* | 13.0412 | 43.8086 | 4.4 | 3.9 | 2.8 | 3.2 |
| FAUZ* | 13.5540 | 44.0564 | -1.3 | -1.3 | -0.4 | -1.3 |
| MOIE | 13.1235 | 43.5032 | 0.3 | 0.5 | 1.7 | 1.2 |
| PSRO | 12.9184 | 43.9007 | 1.6 | 1.8 | 1.0 | 0.8 |
| SENI | 13.2150 | 43.7076 | 0.7 | 1.3 | 4.4 | 4.1 |

**Table S1.** Linear inversion results. Comparison between observed and modelled coseismic displacements (planar components) at GNSS stations.

**References**

1. Palano, M. et al. Geopositioning time series from offshore platforms in the Adriatic Sea. *Scientific data*, ***7***(1), 1-16. (2020).
2. Herring, T.A.; King, R.W.; Floyd, M.A.; McClusky, S.C. Introduction to GAMIT/GLOBK, Release 10.7; Massachusetts Institute of Technology: Cambridge, UK, 2018. Available online: www-gpsg.mit.edu.
3. Fantoni, R., & Franciosi, R. Tectono-sedimentary setting of the Po Plain and Adriatic foreland. *Rendiconti Lincei*, **21**, 197-209. (2010)
4. Atzori, S., Manunta, M., Fornaro, G., Ganas, A., & Salvi, S. Postseismic displacement of the 1999 Athens earthquake retrieved by the Differential Interferometry by Synthetic Aperture Radar time series. *Journal of Geophysical Research: Solid Earth*, **113**(B9). (2008) <https://doi.org/10.1029/2007JB005504>
5. DISS Working Group. Database of Individual Seismogenic Sources (DISS), Version 3.3.0: A compilation of potential sources for earthquakes larger than M 5.5 in Italy and surrounding areas. Istituto Nazionale di Geofisica e Vulcanologia (INGV). <https://doi.org/10.13127/diss3.3.0> (2021).
